# Analysis of Factors Associated With Length of Stay of Opioid-Related Emergency Department Visits

**DOI:** 10.7759/cureus.16213

**Published:** 2021-07-06

**Authors:** Keshab Subedi

**Affiliations:** 1 iREACH, ChristianaCare Health Systems, Wilmington, USA

**Keywords:** emergency department, length of stay, opioid abuse, substance use disorder (sud), accelerated failure time model

## Abstract

Introduction and Objective: Emergency department (ED) length of stay (LOS) is an important indicator of the quality of care in ED and is associated with patients’ outcomes and healthcare costs. However, there is limited data on how the patient characteristics affect the ED LOS of opioid-related visits. This study aims to identify and quantify the effect of patient-related characteristics on LOS of opioid-related ED visits.

Methods: This is a retrospective analysis of electronic health records (EHR) of patients with diagnoses of opioid abuse. The study included patients with a diagnosis of opioid abuse who visited the ED at Christiana Care Hospital from January 1, 2017, to December 31, 2018 (N=5,661). The opioid-related visits were identified using ICD-10 diagnosis codes. We used accelerated failure time (AFT) models, a time-to-event analysis approach to evaluate the relationships of different patient characteristics with ED LOS.

Results: The mean age of the study population was 39 years. The study population had 40% female, 20% Black/African American, and 5% Hispanic or Latino. The prevalence of co-use of cocaine and co-use of alcohol was 11%, and 9%, respectively. Also, 58% had mental health comorbidity, and 1% were homeless. The distribution of ED LOS was right-skewed with a median of 4.3 (IQR: 2.6, 6.8). Co-use of alcohol (time ratio, TR: 1.31, CI: 1.23-1.40), co-use of cocaine (TR: 1.18, CI: 1.11-1.25), the presence of mental health comorbidity (TR: 1.05, CI 1.01-1.09), and homelessness (TR: 1.57, CI: 1.32-1.86) were associated with increased ED LOS.

Conclusions: Co-use of alcohol, co-use of cocaine, homelessness, and mental health comorbidity are associated with the longer LOS of opioid-related ED visits.

## Introduction

Delays in emergency department (ED) patient disposition lead to poor patient outcomes and increased healthcare costs. ED length of stay (LOS) has been used as one of the indicators of quality of care in the ED [1] and is reported to be both the cause and result of ED crowding forming a vicious cycle [2]. Health policy-makers and healthcare providers have instituted a variety of metrics, such as door-to-diagnostic time, door to treatment time, and ED arrival to ED departure time to evaluate and improve the quality-of-care delivery in the ED [3]. Due to the opioid epidemic in the United States, a substantial proportion of ED visits are opioid-related. The rate of opioid-related ED visits increased from 87 per 100,000 population in 2008 to 242 per 100,000 population in 2016 [4]. Further, substance abuse has been attributed to increased resource utilization and longer ED LOS [5-7]. Therefore, it is important to understand the determinants of the LOS of opioid-related visits, especially given that the ED boarding, the practice of waiting in the ED for transition to an inpatient setting or to another facility, has become another pervasive public health problem [8]. Numerable studies have explored the factors associated with ED LOS, focusing both on the entire ED population [9-11], and specific patient’s population such as surgical critical care patients [12,13], psychiatric and mental health patients [14-17], and ethanol intoxicated patients [18]. However, there is limited data on how the patient characteristics affect the LOS of opioid-related ED visits. We hypothesize that certain patient characteristics like co-use of alcohol, co-use of cocaine, presence of mental health comorbidity, and homelessness might increase LOS of opioid-related ED visits. This study aims to describe patients with opioid-related visits in ED of a Level 1 trauma center and to identify and quantify the effect of patient-related characteristics on LOS of opioid-related ED visits. A better understanding of the determinants of LOS of opioid-related ED visits could help identify patient populations who require special intervention or resources. These findings could also help prevent protracted ED stay while improving both the quality of care and patients’ outcomes.

## Materials and methods

Study design and setting

This is a retrospective analysis of electronic health records (EHR) of the patients with a diagnosis of opioid abuse who visited the ED at Christiana Care Hospital from January 1, 2017, to December 31, 2018. Christiana Care Health System is one of the largest healthcare providers in the mid-Atlantic with a Level 1 trauma center serving all of Delaware and parts of Pennsylvania, Maryland, and New Jersey. This study was approved by the Christiana Care Health System’s institutional review board.

Data and data preparation

The analysis dataset was extracted from the EHR system of the Christiana Care Health System. Opioid-related visits were identified using ICD-10 diagnosis codes: F11.x, R78.1, and T40.0x to T40.6x [19]. This definition encompasses both prescription and nonprescription abuse of opioids, including heroin, carfentanil, fentanyl, and methadone19. This study included only those visits that ended with direct discharge from ED. The ED visits in which the patient died, was kept in 23-hour observation, admitted to an inpatient or another facility were excluded from the analysis. The visits in which patients left against medical advice were also excluded from the analysis.

The demographic information in the data included patients’ age, sex, race, and ethnicity. We used the Elixhauser algorithm [20] based on ICD-10 codes to identify the major comorbidities including AIDS, diabetes, hypertension, liver disease, hypothyroidism, neurological disease, pulmonary disease, anemia deficiency, arthritis, blood loss, and coronary artery disease. Other variables included the patients’ mode of arrival, patients’ primary insurance type, triage severity index, and homelessness. The mode of ED arrival was regrouped in to two groups - “ambulance arrival” if the mode of arrival was coded as ambulance arrival, and “other” if the mode of arrival was other than ambulance arrival including missing. The ED arrival shift was computed based on ED registration time. We also identified if the patient was co-using other substances, such as alcohol, and cocaine during the visit, in addition to opioid using ICD-10 diagnosis codes [21]. The LOS was defined and computed as the time from registration in the ED to discharge from the ED.

Statistical analysis

Summary statistics were calculated for patient’s demographic and clinical variables. For categorical variables proportions were calculated, and for numerical variables mean and standard deviation are reported. We defined discharge from the ED as an event of interest and followed time-to-event analysis approaches. We plotted the Kaplan-Meir curve of survival (still being in the ED) stratified by patients’ age category, sex, race, ethnicity, insurance type, the presence of mental health comorbidity, co-use of cocaine, co-use of alcohol, and homelessness. The differences in the Kaplan-Meir curves were tested using the log-rank test. The multivariable accelerated failure time (AFT) models were used to evaluate the effect of patients’ demographic and clinical characteristics on the LOS of ED visits. The AFT model is a type of survival analysis that directly models the log of time to an event as a function of a vector of model covariates [22]. We fitted the AFT models with exponential, log-normal, gamma, and Weibull distribution. The best fit model was selected using a likelihood-ratio test, and Akaike information criteria (AIC). The variables included in the models were guided by the clinical relevance rather than preliminary univariate analysis or arbitrary threshold P-value. The independent variables included in the models were age, sex, race, ethnicity, insurance type (private/ Medicare/Medicaid/ no insurance), the presence of mental health comorbidity (yes/no), arrival shift (morning/day/evening), weekend arrival (yes/no), co-use of alcohol (yes/no), co-use of cocaine (yes/no), and homelessness (yes/no). For ease of interpretation, the estimates from the AFT model are presented as time ratio (TR) calculated as eβ, where β is the regression coefficient from the AFT model. The TR, also referred to as an acceleration factor, can be interpreted as a ratio of LOS of the patients at a given level of a categorical variable to the patients at the reference level of the categorical variable. For numerical variables, the TR is the ratio of LOS corresponding to one unit change in the numerical variable.

Three percent of the visits had at least one of the variables of interest missing. The visits with a missing value(s) were removed from analysis using a pairwise deletion approach resulting in an available case analysis, where cases were excluded from only operations in which data were missing on a variable that was required. Statistical analyses were done using SAS 9.4® (SAS Institute, Cary, NC, USA) and the graphs were plotted using R.

## Results

Patient characteristics

We identified 5661 ED visits with a diagnosis of opioid abuse during the study period. The mean age of the patients was 39 years (SD:12.77) where 40% of the patients were women, 23% were Black/African American, and 5% were Hispanic or Latino. The majority of the patients (57%) had Medicaid coverage, followed by private insurance (15%), and Medicare (10%), while 12% had no insurance coverage. The majority were classified as level 2 (44%) and level 3 (42%) in the triage severity index (Table 1). The most prevalent comorbidities were depression (52%) followed by alcohol use disorder (39%), chronic pulmonary disease (36%), and psychosis (35%).

**Table 1:** Characteristics of the study population. The values are count and percentage unless otherwise noted. ED - emergency department

Variable	Total patients (N=5,661)
Age [mean (SD)]	39 (12.77)
Sex: Female	2,246 (39.67)
Race: Black/African American	1,305 (23.05)
Ethnicity: Hispanic or Latino	262 (4.63)
Insurance: Medicaid	3,527 (56.84)
Insurance: Medicare	602 (10.63)
Insurance: Private	832 (14.70)
No Insurance Coverage	700 (12.37)
ED arrival mode: Ambulance	2,899 (51.21)
Weekend Arrival	1,532 (27.08)
Arrival Shift: Day	2,225 (39.30)
Arrival Shift: Evening	2,378 (42.01)
Arrival Shift: Night	1,058 (18.69)
Triage Severity Index: 1	64 (1.23)
Triage Severity Index: 2	2,470 (43.86)
Triage Severity Index: 3	2,403 (42.45)
Triage Severity Index: 4	628 (11.09)
Triage Severity Index: 5	96 (1.70)
Co-use of Alcohol	492 (8.69)
Co-use of Cocaine	898 (10.56)
Mental Health Comorbidity	3,310 (58.47)
Homeless	65 (1.15)

Results from univariate analysis of the ED LOS

**Figure 1:**
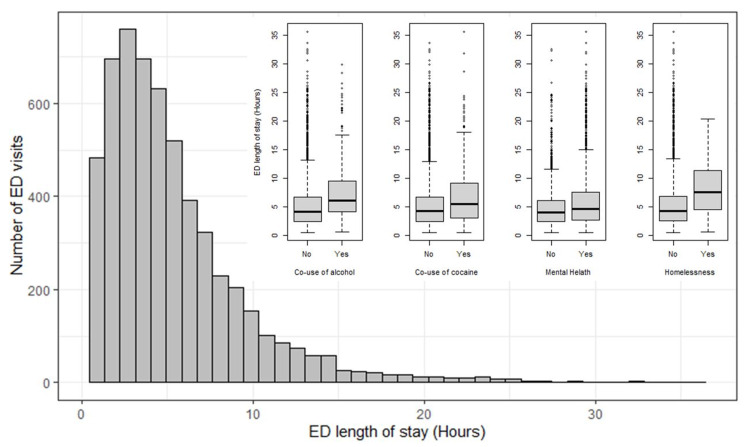
Distribution of the length of stay of opioid-related ED visits. The box plots show the comparisons of ED length of stay of the patients with and without co-use of alcohol, co-use of cocaine, mental health comorbidity, and homelessness (from left to right). ED - emergency department

**Figure 2:**
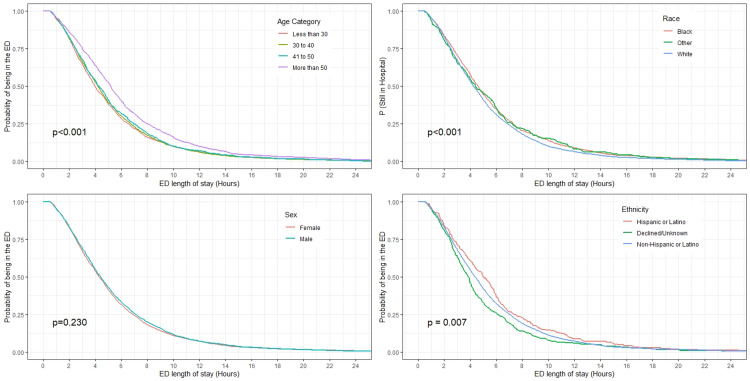
Kaplan-Meir curves of the probability of still being in the ED as a function of time for the strata of age category, race, sex, and ethnicity. The p-values were computed using the log-rank test. The curves are truncated at the length of stay of 24-hour for better visual presentation. ED - emergency department

**Figure 3:**
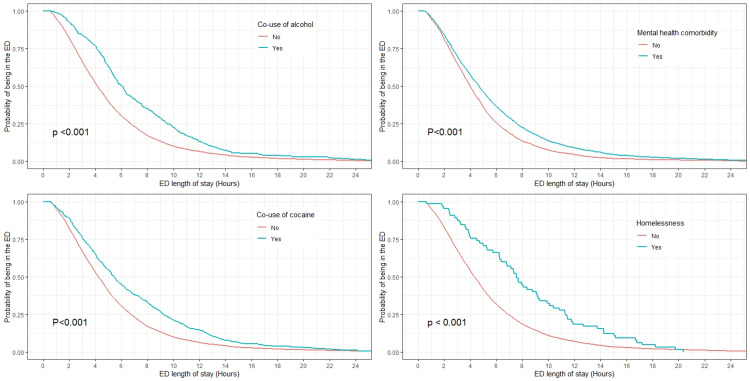
Kaplan-Meir curves of the probability of still being in the ED as a function of time for the strata of co-use of alcohol, co-use of cocaine, the presence of mental health comorbidity, and homelessness. The p-values were computed using the log-rank test. The curves are truncated at the length of stay of 24-hour for better visual presentation. ED - emergency department

The distribution of ED LOS was right-skewed with a median of 4.3 hours (IQR: 2.6, 6.9) (Figure 1). The probability of still being in the ED after one hour, three hours, and six hours were 95%, 68%, and 32% respectively. The Kaplan-Meir curves of the probability of still being in ED stratified by age category, sex, race, ethnicity, co-use of alcohol, co-use of cocaine, the presence of mental health comorbidity, and homelessness are presented in Figures 2, 3. The survival plots plotting the probability of still being in ED were significantly different for the strata of age category (p<0.001), race (p<0.001), ethnicity (p=0.007), co-use of alcohol (p<0.001), co-use of cocaine (p<0.001), the presence of mental health comorbidity (p<0.001), and homelessness (p<0.001) with older patients, Black/African American, Hispanic or Latino, co-use of alcohol, co-use of cocaine, the presence of mental health comorbidity, and homeless having prognosis of longer ED LOS (Figures 2, 3). The median LOS of visits with and without co-use of alcohol was 6.1 vs 4.2 hours. Similarly, the median LOS was 5.5 vs 4.2 hours for visits with and without co-use of cocaine, 4.7 vs 3.9 hours for visits with and without mental health comorbidity, and 8.3 vs 5.3 hours for visits with and without homelessness. The median LOS for visits with co-use of both alcohol, and cocaine was 8.4 hours.

Results from multivariable AFT models of ED LOS

The AFT models with a Gamma distribution were found to be the best fitting models based on the goodness-of-fit test using the likelihood-ratio statistic and AIC. The results from the AFT model are presented in Table 2. The results showed that co-use of cocaine, co-use of alcohol, mental health comorbidity, homelessness, number of comorbidities, and triage index were significantly associated with the LOS of the opioid-related ED visits. Similarly, patients’ demographic factors such as age, sex, race, and ethnicity were also significantly associated with the ED LOS. The patients' ED arrival shift was also significant. The visit day (weekday vs weekend) and patients’ insurance type were not associated with the ED LOS.

The patients with co-use of alcohol had 31% longer LOS compared to the patients who were not diagnosed with alcohol abuse during the visit (TR=1.31, p<0.001). Similarly, the patients with co-use of cocaine during the visit had an 18% longer LOS compared to those without co-use of cocaine (TR=1.18, p<0.001). The patients with mental health comorbidity had 5% longer ED LOS compared to the patients without mental health comorbidity. Homelessness was associated with a 57% longer LOS (TR=1.57, p<0.001). Similarly, incremental age (1 year, TR=1.02, p<0.001) female sex (TR=1.06, p=0.001), Black/African American race (TR=1.07, p<0.001), Hispanic or Latino ethnicity (TR=1.11, p=0.048) were associated with an increased LOS of the ED visits.

**Table 2:** Results from the accelerated failure time (AFT) model of length of stay of opioid-related ED visits. ED - emergency department

Variable	Time ratio (TR) ( 95% CI of TR)	P-value
Age	1.02 (1.01, 1.04)	0.010
Female vs Male	1.06 (1.02, 1.10)	0.001
Black/African American vs White	1.07 (1.02, 1.12)	0.002
Hispanic or Latino vs Non-Hispanic or Latino	1.11 (1.01, 1.23)	0.048
Medicaid vs Commercial Insurance	0.95 (0.90, 1.01)	0.151
Medicare vs Commercial Insurance	1.04 (0.95, 1.13)	0.328
Weekend vs Weekday Arrival	0.97 (0.94, 1.02)	0.295
Day Shift vs Night Shift Arrival	0.96 (0.92, 1.01)	0.163
Evening Shift vs Night Shift Arrival	1.04 (0.99, 1.09)	0.088
Triage Severity Index	0.72 (0.70, 0.74)	<0.001
Elixhauser Comorbidity Count	1.02 (1.01, 1.03)	<0.001
Co-use of Alcohol	1.31 (1.23, 1.40)	<0.001
Co-use of Cocaine	1.18 (1.11, 1.25)	<0.001
Mental Health Comorbidity	1.05 (1.01,1.09)	0.028
Homeless	1.57 (1.32, 1.86)	<0.001

Limitations

This study has several limitations. The data for the study came from a single hospital system so the results may not be generalizable to other hospitals. The list of covariates evaluated to include in the models was not exclusive and there might be other possible factors associated with LOS, which were not included in the model. Previous studies have reported ED occupancy [23-25], and service processes like laboratory tests and imaging [26] to be significant predictors of LOS in ED. Also, the analysis does not control for visits related to opioid overdose and opioid withdrawal, which may have different implications on the ED LOS.

## Discussion

Co-use of alcohol and co-use of cocaine were significantly associated with increased ED LOS of opioid-related ED visits. Patients with co-use of alcohol and patients with co-use of cocaine spent 31% and 18% longer times in ED respectively compared to the patients without that co-usage. Co-use of alcohol and co-use of cocaine might have increased the ED LOS due to additional health complexities related to multiple drug usage. These patients may also have to go through additional laboratory tests and procedures while in ED. To the knowledge of the author, there is no study that reports on how co-use of alcohol and co-use cocaine among patients with opioid abuse affects ED LOS and hospital utilization in general. However, past studies have reported an association of alcohol use and an increased ED LOS in mental health patients [15], minimally injured patients [27], and psychiatric patients [28]. A previous study reported that psychiatric patients with a negative toxicology screen result stayed an average of 12 hours (10.6 to 13.5), whereas patients who presented with any serum alcohol level greater than zero, with or without other substances, had average ED stays of 14.5 hours (12.3 to 17.2) [28]. These results indicate that alcohol abuse is an important factor associated with the increased LOS irrespective of patient populations.

Homelessness was associated with an increase in ED LOS with a TR of 1.57, which corresponds to a 57% longer LOS compared to patients who were not homeless. Previous studies have reported similar results but in different patient populations. Homelessness was reported to be associated with increased LOS by 45 % in psychiatric patients [29], and by 7% in patients with a history of substance use disorder (SUD) [5]. The relationship between longer ED LOS and homelessness has been implicated in higher rates of medical comorbidities and mental health disorders among homeless patients [30]. To note, the analysis in this study adjusted for a number of comorbidities, and the presence of mental health comorbidity. The longer LOS of homeless patients could be related to delays in the discharge of homeless patients due to a lack of immediate safe disposition and post-discharge shelters.

Sex was another factor associated with ED LOS with the females spending longer time in the ED compared to the males. The results showed a TR of 1.09 for females to males, which corresponds to a 9% longer LOS for females compared to males. Results from previous studies on the effect of sex on ED LOS are not consistent and varied by patient population. A previous study analyzing the LOS of the total ED population reported similar results with females spending significantly longer time in ED than males, but the effect size was much smaller [10]. However, in a population-based study in Ontario, Canada, sex was reported not to be a significant determinant of prolonged ED stay [13]. Further, we find a racial and ethnic disparity on the ED LOS with Black/African American, and Hispanic or Latino spending 7% and 11% longer time in the ED compared to their White, and Non-Hispanic or Latino counterparts. These findings support previously documented racial and ethnic disparities in timely receipt of ED care and LOS in different patient populations [31-33]. Bekmezian et. al explained similar ethnic and racial disparity in ED LOS in the pediatric population to be possibly caused by language barrier [31], and this might be a potential factor in opioid-related visits. However, we need prospective studies, and root-cause analyses to better explain these racial and ethnic disparities.

## Conclusions

This study is unique in exploring the effect of patient’s characteristics on LOS of opioid-related visits. We find that female sex, Black/African American race, Hispanic or Latino ethnicity, co-use of alcohol, co-use of cocaine, homelessness and the presence of mental health comorbidity are associated with increased ED LOS. These results could be helpful to physicians and patients to better anticipate an individual’s LOS and could help administrators devise tailored interventions and processes to reduce protracted ED stays.
